# Development of Natural Active Agent-Containing Porous Hydrogel Sheets with High Water Content for Wound Dressings

**DOI:** 10.3390/gels9060459

**Published:** 2023-06-03

**Authors:** Thanyaporn Pinthong, Maytinee Yooyod, Jinjutha Daengmankhong, Nantaprapa Tuancharoensri, Sararat Mahasaranon, Jarupa Viyoch, Jirapas Jongjitwimol, Sukunya Ross, Gareth M. Ross

**Affiliations:** 1 Biopolymer Group, Department of Chemistry, Faculty of Science, Naresuan University, Phitsanulok 65000, Thailand; thanyapornpi62@nu.ac.th (T.P.);; 2 Biopolymer Group, Department of Chemistry, Center of Excellence in Biomaterials, Faculty of Science, Naresuan University, Phitsanulok 65000, Thailand; 3 Department of Pharmaceutical Technology, Faculty of Pharmaceutical Sciences and Center of Excellence for Innovation in Chemistry, Naresuan University, Phitsanulok 65000, Thailand; jarupav@nu.ac.th; 4 Department of Medical Technology, Faculty of Allied Health Sciences and Center of Excellence in Biomaterials, Faculty of Science, Naresuan University, Phitsanulok 65000, Thailand

**Keywords:** porous hydrogels, wound dressings, Manuka honey, surface absorption, 2-acrylamido-2-methyl-1-propane sulphonic acid

## Abstract

This work was concerned with the fabrication of a porous hydrogel system suitable for medium to heavy-exudating wounds where traditional hydrogels cannot be used. The hydrogels were based on 2-acrylamido-2-methyl-1-propane sulfonic acid (AMPs). In order to produce the porous structure, additional components were added (acid, blowing agent, foam stabilizer). Manuka honey (MH) was also incorporated at concentrations of 1 and 10% *w*/*w*. The hydrogel samples were characterized for morphology via scanning electron microscopy, mechanical rheology, swelling using a gravimetric method, surface absorption, and cell cytotoxicity. The results confirmed the formation of porous hydrogels (PH) with pore sizes ranging from ~50–110 µm. The swelling performance showed that the non-porous hydrogel (NPH) swelled to ~2000%, while PH weight increased ~5000%. Additionally, the use of a surface absorption technique showed that the PH absorbed 10 μL in <3000 ms, and NPH absorbed <1 μL over the same time. Incorporating MH the enhanced gel appearance and mechanical properties, including smaller pores and linear swelling. In summary, the PH produced in this study had excellent swelling performance with rapid absorption of surface liquid. Therefore, these materials have the potential to expand the applicability of hydrogels to a range of wound types, as they can both donate and absorb fluid.

## 1. Introduction

Hydrogels are chemically or physically cross-linked hydrophilic polymer networks that are able to absorb and retain a large amount of water or biological fluids. They are often used in tissue engineering [1], drug delivery [2,3,4], and biomedical devices (e.g., wound dressings and contact lenses) [5,6] due to their biomimetic properties and high water content. Hydrogel wound dressings are important medical devices that can be used for a wide range of wounds such as shallow and deep open wounds (pressure sores, leg ulcers, surgical and malignant wounds, partial thickness burns, scalds, and lacerations) that may be located on hard-to-fit locations on the body (such as joints, hands, and face) [7,8].

However, one area where hydrogel wound dressings cannot currently be used is medium and heavy-exudating wounds, which usually require foam dressings (e.g., polyurethane) [9]. The reason for this is that hydrogels, despite their capacity to absorb substantial amounts of liquid, typically experience a delay in swelling at the outset of application. During this delay, the hydrogel network undergoes a process of structural reorganization where the polymer chains separate from one another, generating gaps that permit water to enter the hydrogel [3,10]. This limitation means that traditional hydrogel wound dressings cannot be used for medium and heavy-exudating wounds. Nevertheless, by incorporating a porous structure into the hydrogel, it is thought that the hydrogel can become capable of effectively absorbing higher amounts of exudate from wounds with medium and heavy exudation. This is demonstrated by a group of hydrogels termed “superporous hydrogels” (SPH) that have been shown to rapidly absorb large amounts of water due to the presence of interconnected microscopic pores [11].

SPHs exhibit this behavior because of their porous configuration, which provides a significantly larger surface area and shorter diffusion distance compared to conventional hydrogels. These gels can have a similar composition to conventional hydrogels but contain a system for producing a porous structure during the polymerization process. A variety of methods exist to generate macroporous hydrogel structures, such as emulsion [12], freeze drying [13,14,15], high internal phase emulsion (HIPE) [16], water in oil emulsion templates [17], and the gas blowing technique [18].

Numerous polymers can be utilized to create hydrogel wound dressings, such as poly(vinyl pyrrolidone), poly(vinyl alcohol), poly(acrylic acid), polyesters, 2-acrylamido-2-methylpropane sulfonic acid (AMPS), and its sodium salt [19]. AMPS-based polymers are highly desirable due to their inherent advantages. The presence of a sulfonate group in AMPS resembles the glycosaminoglycan present in the skin’s extracellular matrix, which plays a key role in maintaining and providing moisture to the body. This property makes AMPS hydrogels act as synthetic counterparts of proteoglycans. Moreover, AMPS has been proven to accelerate epithelialization, alleviate pain, and stimulate bioactivity in ulcerated wounds [19,20,21,22]. This has enabled AMPS to be used for several hydrogel wound dressings and injectable hydrogels. For example, AMPS has been combined with other polymers such as poly(ε-caprolactone) diacrylate and carboxymethyl chitosan to form AMPS-containing wound dressing hydrogels [23]. Injectable sulfonate-containing hydrogels with AMPS have also been produced from thiol-containing copolymers reacted with a four-arm acrylamide-terminated poly(ethylene glycol) via a thiol-ene click reaction [24]. The aforementioned benefits serve as the underlying justification for the utilization of AMPs in this study.

Many hydrogel wound dressing materials also benefit from the incorporation of natural healing aids. There are several options to consider, such as Aloe vera [25], Centella asiatica [26], Echinacea purpurea [27], and Manuka honey [28,29,30]. Manuka honey (MH) is a mono-floral honey obtained from the Leptospermum scoparium tree native to New Zealand. It has been shown to stimulate angiogenesis, macrophages, and wound epithelialization [31]. In addition, it can provide nutrition components during the wound healing process [32,33] and exhibits inflammation modulation, thereby reducing the inflammation phase and promoting wound healing [33]. Interestingly, MH possesses antibacterial and antibiofilm affects, a low pH range (3.2–4.5), and degradation to hydrogen peroxide which contributes to bacterial death [34,35]. In terms of manufacturing, MH also influences the viscosity of the system, which can help control how the components are mixed.

In this study, we investigated the potential of gas-blown porous hydrogel sheets, which is a novel approach to enhance the absorption properties of the hydrogels for treating medium to heavy-exudating wounds. The primary goal was to develop hydrogels that exhibit not only high absorption capacity but also excellent mechanical stability. The gas-blown porous hydrogel sheets were fabricated through the synthesis of hydrophilic monomer and 2-acrylamido-2-methyl-1-propane sulfonic acid sodium salt (AMPs), a foaming agent, and with the incorporation of MH. Non-porous hydrogels (NPH) were compared to porous hydrogels (PH) by observing the appearance and rheological mechanical properties of the gels. Then, the absorption properties of the gels were assessed in terms of bulk swelling capacity and a novel technique developed to measure the real-time surface absorption. Two different concentrations of MH were added to the system (1 and 10% *w/w*), and a range of material characterization techniques was used to study how the incorporation of MH affected the performance of the gels. Additional assessments were conducted to examine the morphology of the gels and cell viability to ensure that the materials were non-toxic. These properties are crucial for ensuring that the material can effectively as a wound dressing for all wound types, including those with heavy exudate.

## 2. Results and Discussion

**Figure 1:**
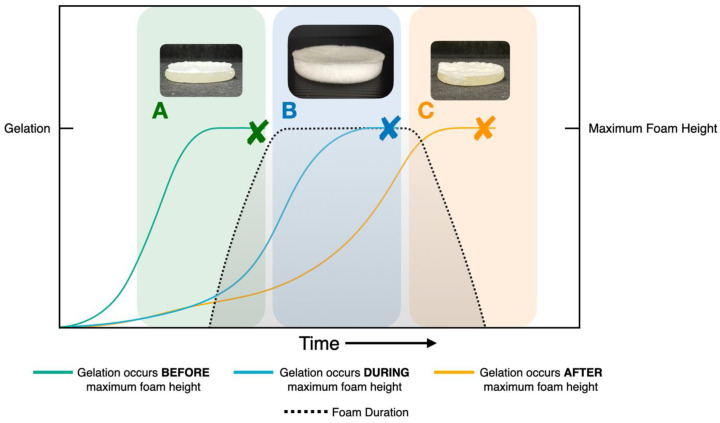
Relationship between gelation time and maximum foam height. (**A**) Gelation occurs before foam reaches maximum height, (**B**) gelation occurs during maximum foam height, and (**C**) gelation occurs after maximum foam height.

The fabrication of porous hydrogels is achieved when the foaming agent (sodium bicarbonate (BA)) is decomposed by an acid (methacrylic acid (MAA)) during gelation to produce CO_2_. The hydrogels are polymerized around the gas bubbles, producing a porous structure. Methacrylic acid (MAA) was selected to be incorporated into the hydrogel structure during the polymerization step. This fabrication of porous hydrogels (PH) requires the balance of gelation time and gas blowing formation to obtain the optimal porous structure. Figure 1 demonstrates that when gelation occurs before or after the maximum foam height, the resulting hydrogels exhibit a two-layered structure. For example, for the gel in Zone A, the concentration of redox initiators was increased to 2 M, resulting in rapid gelation. This caused the gel to form in two layers because the foam reaction was still at the early stages when the gelation was completed. This shows that an excessively fast gelation time leads to the formation of a two-layered system, which is not desirable for creating homogeneously porous hydrogels. In Zone C, the concentration of redox initiators was decreased to 0.5 M. This resulted in a slower gelation time, which allowed for the foam to reach its maximum height prior to dissipating before complete gelation. Consequently, since the polymer matrix could not trap the foam before the gelation was complete, the resulting gel also did not possess a homogeneously porous structure. This suggests that gelation times that are too slow to occur during the maximum foam height may also not be suitable for creating homogeneously porous hydrogels. However, when gelation takes place at the ideal time (Zone B), which corresponds to the peak foam height, the resulting hydrogel exhibits a homogeneous porous structure.

Initially, we compared the properties and differences between non-porous hydrogels (NPH) and porous hydrogels (PH) without honey (Section 2.1). The confirmation of the porous structure was examined, as well as how it influences the mechanical strength and swelling behavior of the gels. Additionally, a surface absorption method was employed that was more closely related to the future application of the material in absorbing wound fluid at the surface. After confirming that the porous hydrogel system was viable, the addition of MH was studied. In preliminary studies, the %MH was varied at the following compositions: −1%, 2%, 5%, 10%, 15%, and 25%, with 1 and 10% exhibiting homogeneous porous structures, while the others altered the balance between gelation and foam quality to produce dual-layered systems. Therefore, concentrations of 1 and 10% *w*/*w* were studied further (Section 2.2).

### 2.1. Comparisons of Non-Porous Hydrogels (NPH) and Porous Hydrogels (PH)

**Figure 2:**
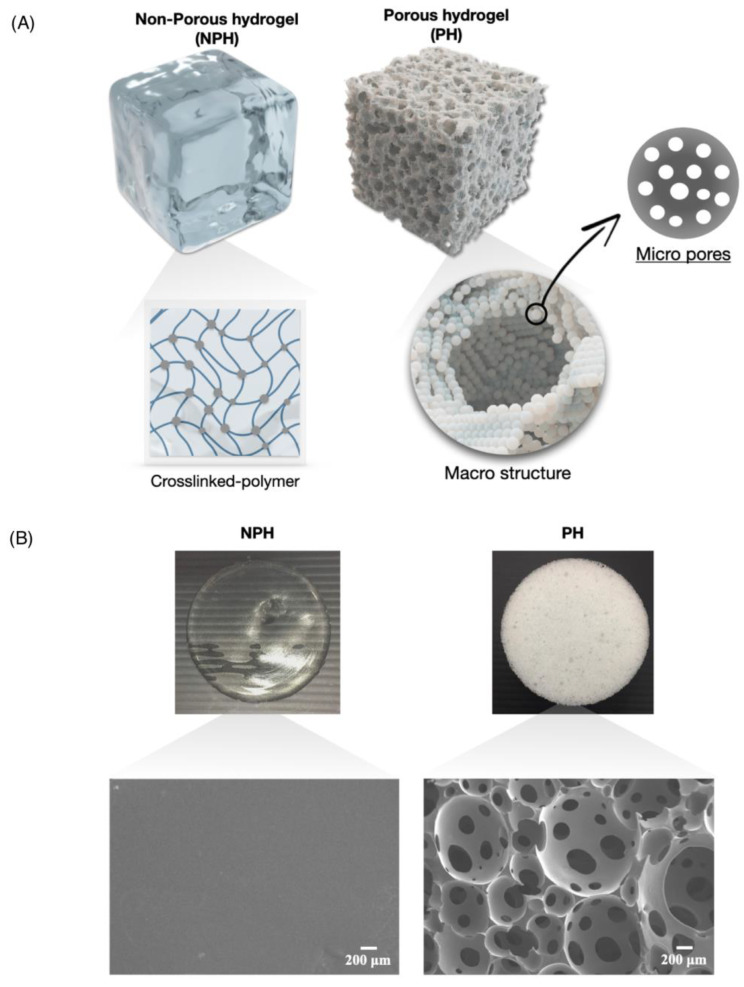
A schematic of three-dimensional non-porous hydrogel (NPH) and porous hydrogel (PH) (**A**). Visualization and scanning electron microscopy micrographs of non-porous hydrogel (NPH) and porous hydrogel (PH) structures (**B**).

Figure 2 shows a graphical representation (Figure 2A) and visualization/scanning electron microscopy (SEM) micrographs (Figure 2B) of the NPH and PH. There was a marked difference between the two hydrogels, with the NPH presenting a clear appearance, while the PH was opaque. This opaque appearance was due to the porous structure of the PH, which is schematically show in Figure 2A. To validate the morphology depicted in the schematic, SEM was employed to examine the morphologies of both samples. Upon analyzing the SEM samples, it was observed that the NPH had a smooth and unblemished surface. On the other hand, the PH sample exhibited both macro structures formed by “polymer droplets” and microstructures resulting from the gas blowing process.

**Figure 3:**
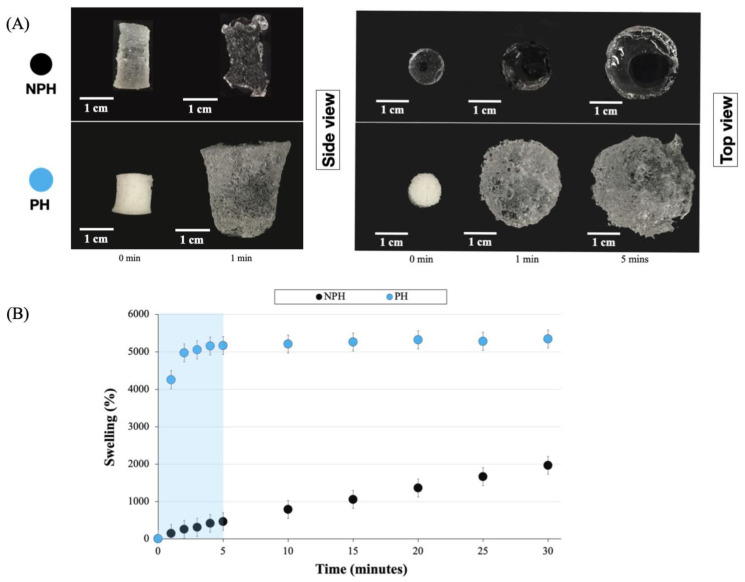
Bulk swelling in water and appearance of NPH and PH at 0 and 1 min respectively. (**A**) Side view and top view. (**B**) Percentage swelling of NPH and PH.

The bulk swelling behavior of NPH and PH samples is presented in Figure 3. Figure 3A shows the appearance of the gels before immersion in water at 0 min, after 1 min, and after 5 min. Both the NPH and PH displayed a significant increase in size, swelling from their original 10 mm diameter to 24 mm and 34 mm, respectively, after 5 min. Figure 3B shows the swelling ratios calculated using Equation (1) over a period 30 min. The weight increase for both samples was greater than 1000%, with the PH reaching over 5000%. This value was reached in less than 2 min, with a more consistent % swelling reached after only 5 min. In contrast, the swelling of the NPH samples showed a more linear increase over the entire 30 min. The main difference between the two systems was that the pores present in the PH facilitated faster initial water absorption through capillary action. Another notable observation was that the NPH sample still exhibited considerable swelling, reaching a value of 2–3000% after 24 h. However, this was still considerably lower than the swelling observed in the PH sample. Therefore, the formation of pores in the system led to a twofold increase in swelling.

**Figure 4:**
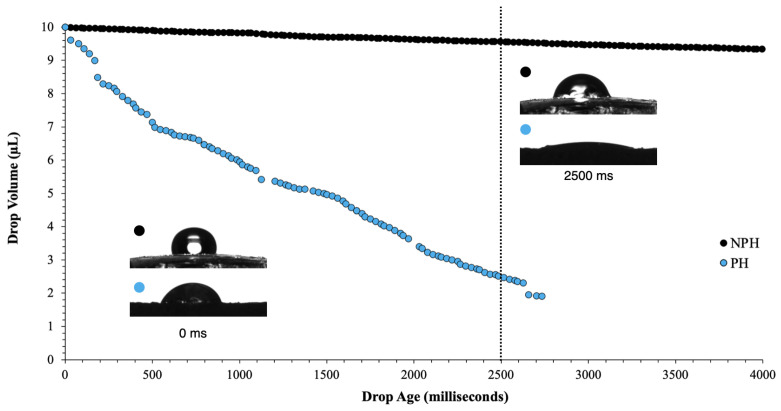
Surface absorption of non-porous hydrogel (NPH) and porous hydrogel (PH). Inset: appearances of droplet at instant of drop placement (0 ms) and after 2500 ms.

Based on the results of the swelling ratio, further investigation was conducted to examine the initial swelling or surface absorption of these gels. Plotting the drop volume (μL) against drop age (milliseconds) allowed for the measurement of surface absorption. The camera recorded at a rate of 36 frames per second, facilitating precise monitoring of the drop volume throughout the absorption process. This enabled a comprehensive real-time assessment of surface absorption for each sample. Figure 4 illustrates the real-time surface absorption of the NPH and PH gels. The PH gel completely absorbed the initial drop volume of 10 μL in less than 3000 ms (3 s), while the NPH gel absorbed water at a considerably slower rate compared to the porous sample. Over the 4000 ms period shown, the NPH sample only absorbed approximately 1 μL. This once again highlights that the NPH experienced a lag in swelling as the polymer chains rearranged themselves to allow water to enter the gel, whereas the pores in the PH enabled water to enter the system immediately upon contact.

### 2.2. Comparisons of Porous Hydrogels (PH) and Porous Hydrogels with Manuka Honey (PH_MH)

**Figure 5:**
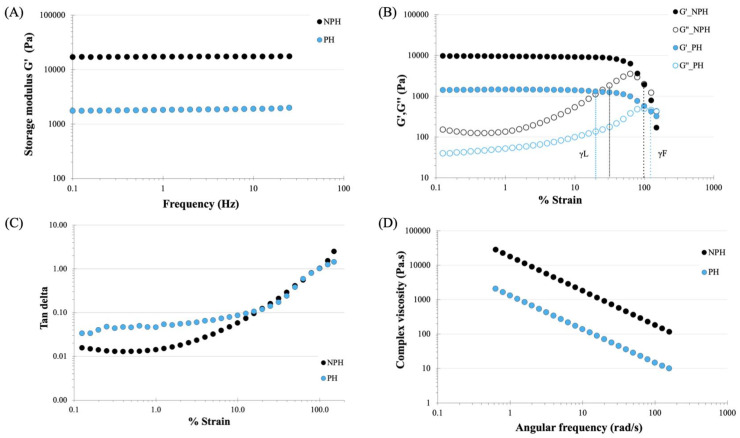
Rheological behavior of NPH and PH under (**A**) frequency sweep—Storage modulus G’ vs. Frequency. (**B**) Strain sweep—Storage and loss modulus G’ and G” vs. % Strain. (**C**) Strain sweep—Tan delta vs. % Strain. (**D**) Complex viscosity vs. Angular frequency.

The mechanical properties of the NPH and PH samples were assessed by measuring the rheological properties, which are presented in Figure 5. The mechanical properties of sheet hydrogels are important in many applications that require handling, such as wound dressings. In regards to wound dressing function, two key requirements can be identified: (1) the material should be able to withstand the expected forces during treatment, and (2) the parameters should not impair normal skin function and preferably promote healing [36]. The samples underwent two different testing regimes: a frequency sweep and a strain sweep. The results from the frequency sweep in Figure 5A show that the NPH had an average storage modulus value of ~17,000 Pa, while the PH has an average value of ~1700 Pa. The frequency-sweep test did not show a crossover between G’ and G” in ether system, and each gel exhibited a modulus was independent of frequency, which is conventional in gel-like systems. The strain sweep in Figure 5B was used to assess the extent of the linear viscoelastic region for each gel. The results indicate linear behavior up to approximately 20% strain (γL) for PH and approximately 30% for NPH, before G’ started to decrease. As the strain increased, crossovers between G’ and G” occurred at 100% (NPH) and 125% (PH) strain (γF), indicating a transition towards a liquid-like response. A material with good cohesive strength should have a high G’ and a low tan delta. The tan delta values of the samples are also presented in Figure 5C, and the results show that both samples have tan delta values below 0.1 at strains below 20%, with the NPH sample exhibiting a lower tan delta value than the PH sample. When the tan delta value is less than 0.1, it indicates that there is good cohesive force. In Figure 5D, the complex viscosity vs. angular frequency plot shows a decreasing linear relationship for both samples, with the NPH having a higher complex viscosity compared to the PH sample. This indicates that there was yield stress in both systems, and that both hydrogels were viscoelastic solids, as they did not flow at rest.

**Figure 6:**
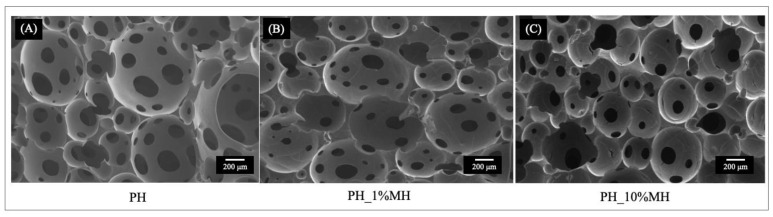
SEM micrographs at 50× magnification of (**A**) PH, (**B**) PH_1% MH, and (**C**) PH_1% MH.

The cross-section structural morphology of the hydrogel samples was observed using SEM. The SEM micrographs showed that when MH was incorporated in the gels, there was a reduction in the average pore size. Figure 6A,B, shows the presence of ‘polymer droplets’ and pores in the structure. In contrast, PH_10% MH (Figure 6C) exhibits a more homogenous polymer structure with similar-sized pores to those in 1% MH. Table 1 presents the averaged pores size and % porosity values. The average pores sizes for PH, PH_1% MH, and PH_10% MH were 108.5 µm ± 46.0, 51.5 ± 24.2, and 50.5 µm ± 11.2, respectively. These pore size values are in line with values observed for other porous hydrogels fabricated using gas foaming techniques, which have been reported within the range of 10–500 µm [37]. The impact of MH concentration on the % porosity within the hydrogel structure shows that the incorporation of 1% MH into the PH resulted in a decrease in porosity from 37.10 ± 34.5% to 21.73 ± 15.5%, whereas the addition of 10% MH increased the porosity to 42.22 ± 11.1%. This indicates that the incorporation of 10% MH caused an increase in the number of pores within the hydrogel structure, leading to a higher overall porosity. The smaller pore sizes and increased porosity resulting from the addition of 10% MH were attributed to the influence of MH on the system. Firstly the viscosity of the system was altered, and secondly, MH is slightly acidic, which affected the size and duration of the gas bubbles formed. Another noteworthy observation was the shape of the PH_10% MH pores, which were highly spherical. This characteristic highlights the enhanced stability of the foam produced by this particular sample.

**Figure 7:**
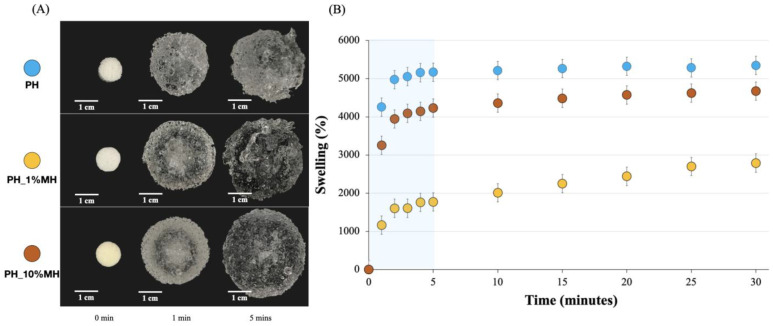
Bulk swelling in water. (**A**) The appearance of PH and PH with 1% and 10% MH at 0, 1, and 5 min, respectively. (**B**) Percentage swelling of PH and PH with 1% and 10% MH.

Figure 7A,B shows the bulk swelling behavior of PH and PH with MH. The results show that the addition of MH resulted in a reduction in the swelling ratio, with 1% MH resulting in a larger decrease compared to 10% MH. The majority of this additional swelling capacity visibly occurred in the initial period (<3 min). After this initial period, the rate at which the gels swelled was similar for all samples. A noteworthy finding was that the gels containing MH seemed to retain their original shape better than the hydrogels that did not contain MH. This was especially visible in the PH_10% MH after swelling for 5 min.

PH_10% MH exhibited a higher percentage of swelling due to its larger surface area and interconnected network within the structure, which contributed to its higher swelling capacity compared to PH_1% MH. Although PH had the most swelling capacity due to its much larger pore size, when considering mechanical properties after swelling, PH with MH had better appearances when compared to PH without MH.

**Figure 8:**
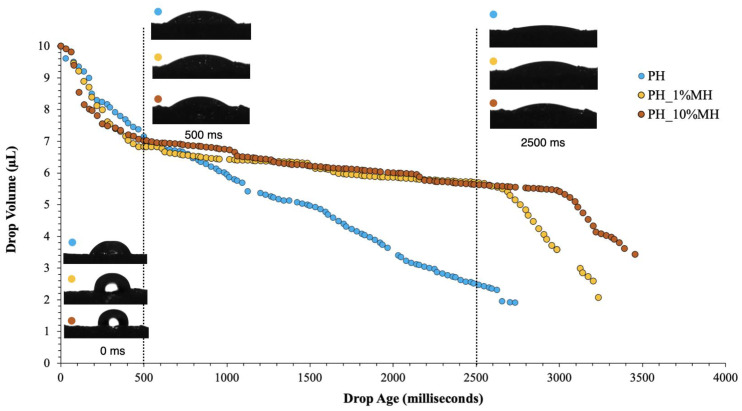
Surface absorption of PH and PH with 1% and 10% MH. Inset: the appearances of droplet at the instant of drop placement (0 ms), after 500 ms, and after 2500 ms.

Figure 8 compares the surface absorption PH samples with 1% and 10% MH. During the initial period (0 to 500 ms), all hydrogels exhibited a very similar and fast absorption rate. After this initial period, the PH samples continued to absorb at a similar rate. However, after 500 ms, the PH_1% MH and PH_10% MH absorption rates decreased to 2700 ms and 3000 ms, respectively. After this time period, the MH samples absorbed the rest of the droplet. One limitation of this technique is that the software could not accurately measure the last microliter of solution. Hence, all sample traces finished before reaching zero. The properties of the hydrogels can be affected by pore size and porosity, with pore size having a significant impact on the movement of water into the gels [38]. In the case of hydrogels containing MH, the pore size was approximately half that of hydrogels without MH, resulting in the distinct absorption behavior observed in these samples. The size of the pores in the hydrogel plays a crucial role in determining the surface absorption capacity. Generally, larger pores have a higher capacity for absorption compared to smaller pores. This is because larger pores provide more surface area and volume for the absorption of fluids or molecules. However, there is an optimal pore size range that balances surface area with the diffusion distance to facilitate efficient absorption. The shape of the pores also affects the surface absorption; pores with irregular shapes or tortuous pathways may hinder the diffusion of fluids or molecules into the hydrogel matrix. On the other hand, well-defined and interconnected pore structures can facilitate the flow and penetration of substances, leading to improved surface absorption. The surface properties of hydrogels significantly impact their ability to absorb substances. These properties include surface charge, surface energy, and surface roughness.

**Figure 9:**
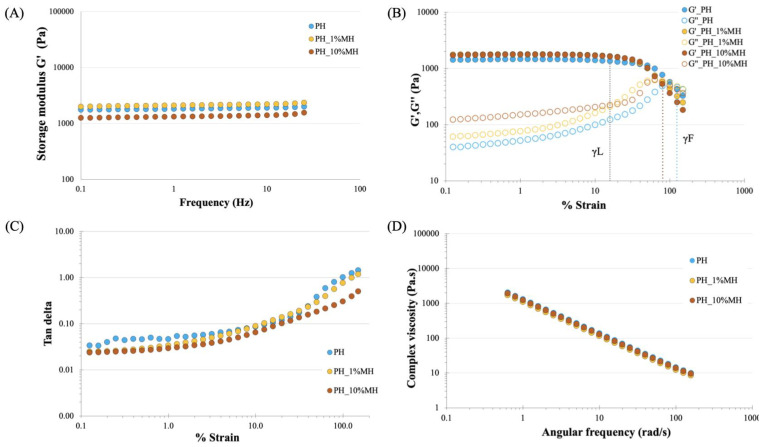
Rheological behavior of PH and PH with 1 and 10% w/w MH. (**A**) Frequency sweep—Storage modulus G’ vs. Frequency. (**B**) Strain sweep—Storage and loss modulus G’ and G” vs. % Strain. (**C**) Strain sweep—Tan delta vs. % Strain. (**D**) Complex viscosity vs. Angular frequency.

Figure 9A,D presents the rheology properties of PH and PH with 1% and 10% MH. These samples were tested using the same parameters as shown in Figure 5. Based on the results shown in Figure 9A, the incorporation of 1% and 10% Manuka honey did not significantly affect the storage modulus during the frequency sweep testing, as all samples exhibited storage modulus values between 1000 and 2000 Pa. Figure 9B illustrates the behavior of PH and PH with 1% and 10% MH during the strain sweep. The results showed that the addition of Manuka honey did not alter the storage modulus of the gels (G’), but there was an increase in the loss modulus (G”) observed in the PH and honey-containing gels (PH_1% MH and PH_10% MH) at 34.66 (PH), 58.62 (PH_1% MH), and 120.09 (PH_10% MH) Pa, respectively. The linear viscoelastic region of the hydrogel (γL) was identical to that of PH, but the crossover of G’ and G” (γF) occurred at approximately 80% strain for the samples containing honey and approximately 100% for the PH samples without honey. The examination of tan delta (Figure 9C), comparing PH with MH at concentrations of 1 and 10% *w*/*w*, revealed a similar pattern. The addition of honey into the PH matrices affected the resulting material’s tan delta values, resulting in a marginally reduced tan delta as the honey concentration increased, while the trend in tan delta remained comparable. Figure 9D shows the complex viscosity plotted against angular frequency for all the samples. The results showed that all three samples exhibited a linear response, further confirming that the samples behaved as viscoelastic solids. The rheological performance of the samples showed that the inclusion of MH into the hydrogel matrix did not reduce the mechanical properties of the hydrogel. The minimal impact of honey on the mechanical properties can be attributed to the fact that honey does not undergo considerable physical crosslinking and exists as an interpenetrating network associated with the water and polymer molecules. However, it is important to note that certain components present in honey, such as 1,2 dicarbonyl compounds (Glyoxal and 3-deoxyglucosulose) and phenolic acids (Gallic acid and 4-methoxyphenylactic acid) [39], have the ability to form hydrogen bonds with the hydrogel network. These components play a role in preserving the strength of the gels and contribute to the decomposition of the sodium bicarbonate blowing agent, ultimately leading to an improved porous structure.

**Figure 10:**
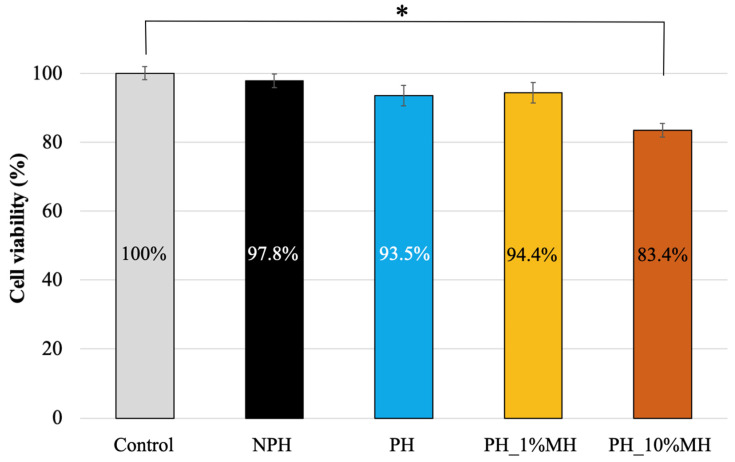
Cytotoxicity test to evaluate cell viability of NPH, PH, PH_1% MH, and PH_10%MH (* *p* < 0.05).

The cytotoxicity of NPH, PH, and PH with 1% and 10% MH was tested using the XTT assay. Fibroblast cells were used to evaluate cell viability over 24 h. Statistical analyses were conducted to indicate significant differences (*p* > 0.05) in cell viability between the samples and the control. Figure 10 shows that all samples exhibited cell viability higher than 80%, with only PH_10% MH demonstrating a cell viability <90% and showing statistical significance compared to the control sample. High Manuka honey content is known to result in a decrease in cell viability due to its hydrogen peroxide and flavonoid content. Moreover, the pH of Manuka honey is acidic, ranging between pH 3.2 and 4.5, which can decrease cell viability. Previous studies have indicated that cytotoxic effects start at 3–5% MH [40]. International guidelines (ISO10993-5, 2009. Biological evaluation of medical Devices, in: Standardization, I.O.f. (Ed.) Part 5: Test for in vitro cytotoxicity, 3 ed. International Organization for Standardization, Geneva, Switzerland) state that a substance is cytotoxic only if it reduces cell viability to less than 70%. All the tested samples exhibited cell viabilities higher than 70% and can therefore be classified as non-toxic.

## 3. Conclusions

This work was concerned with the fabrication method for sheet porous hydrogels (PH) that exhibit exceptional absorption capacity and rapid fluid uptake. The porous hydrogels were synthesized through a delicate balance of three pre-mixtures, resulting in reproducible and reliable production of the gels. Porous hydrogels exhibited higher swelling ratios than non-porous hydrogels (NPH). Additionally, real-time surface absorption analysis revealed significant differences, with porous hydrogels absorbing 10 μL of fluid in 3000 ms, while NPH hydrogels absorbed only 1 μL. To enhance the properties of PH hydrogels for use in wound dressings, Manuka honey (MH) was incorporated into the gel structure at concentrations of 1 and 10% *w*/*w*. The results indicated that MH improved the appearance of the gel, with smaller pores and more linear swelling behavior observed over the first 5 min. The incorporation of MH also improved the mechanical properties of the hydrogels. These improvements were attributed to the inherent properties of MH, which altered the system by increasing viscosity and lowering the pH, enhancing foam production during gelation. Overall, the findings of this study suggest that the synthesized hydrogels have potential for use in wound dressings for medium and heavy-exudating wounds, where conventional hydrogels may not be suitable. When combined with the beneficial properties of MH, the improved properties of PH hydrogels offer a promising avenue for the development of advanced wound dressings with rapid absorption. Future research should focus on modifying the composition in order to achieve the desired pore size of the fabricated porous hydrogels. This can be accomplished by adjusting the balance of surfactants, blowing agents, and acids, while also considering the inclusion of additional components to modify the viscosity. Furthermore, the system can be enhanced by incorporating other active agents, such as silver nanoparticles.

## 4. Materials and Methods

### 4.1. Materials

2–acrylamido-2-methyl-1-propanesulfonic acid sodium salt (AMPs) (Mw = 229.23; 50% wt. in water) (monomer), Methacrylic acid 99% (MAA) (monomer), Di(ethylene glycol) diacrylate 99% (XL) (cross-linker), N,N,N′,N′-Tetramethylethylenediamine (TEMED) (initiator), Ammonium persulphate (APS) (initiator), Poloxamer 407 (Pluronic^®^ F-127) (F127) (surfactant), and Sodium hydrogen carbonate (BA) (blowing agent) were all purchased from Sigma-Aldrich Co. Inc, Singapore, Singapore. Manuka Honey (MH) (86% Manuka pollen content) was purchased from Airborne Honey Ltd., Canterbury, New Zealand. For the cell culture studies, Dulbecco’s modified Eagle’s medium (DMEM), fetal bovine serum (FBS), penicillin–streptomycin, amphotericin B, and 0.25% trypsin–ethylene-diaminetetraacetic acid were purchased from Gibco (Grand Island, NY, USA). The XTT solution (Cell Proliferation Kit II) was supplied by Roche Diagnostics GmbH (Mannheim, Germany). Phosphate-buffered saline (PBS) of pH 7.4 was supplied by KEMAUS, Cherrybrook N.S.W., Australia. Normal human dermal fibroblast (NHDF) cells (Lot no. C-12302, Promocell, Eppelheim, Germany) (1 × 10^5^ cells/well, passage number 6) were provided by the Faculty of Pharmacy, Naresuan University, Phitsanulok, Thailand.

### 4.2. Synthesis of Non-Porous Hydrogels (NPH), Porous Hydrogels (PH), and Porous Hydrogels with Manuka Honey (PH_MH)

#### Synthesis of Hydrogels

Hydrogel samples were prepared using a redox-initiated free-radical polymerization procedure. Three different hydrogel samples were synthesized, including non-porous hydrogels (NPH), porous hydrogels (PH), and porous hydrogels with Manuka honey (PH_MH).

All the hydrogels were prepared using an Ammonium Persulphate (APS) and N, N, N, N,-tetramethyl ethylenediamine (TEMED) redox pair at a concentration of 1M. For the redox pair, APS was added to vial A and TEMED was added to vial B for all samples.

Non-porous hydrogels (NPH) were synthesized by following the composition in Table 2. Briefly, all components were split between two vials (A and B). The NPH gels were comprised of AMPs, deionized water, XL solution, and a redox initiator pair (APS and TEMED). Both vials A and B contained equal amounts of monomer, cross-linker, and deionized water, with 1 M of APS and 1 M TEMED prepared and added in separate vials. Then, each vial was mixed using an orbital shaker for 30 min until a homogenous solution was obtained. In the final step, vials A and B were poured together into a mold, and the mixture was mixed using an overhead mechanical stirrer for 15 s.

For porous hydrogel samples, the monomers (AMPs/MAA), surfactant solutions, cross-linker, blowing agent, and redox pairs were added in three separate sample vials (A, B, and C). For vials A and B, the blowing agent (Sodium hydrogen carbonate) was dissolved in the pre-prepared stock surfactant solution (Poloxamer 407—0.5% F127) and split equally between vials A and B. The monomer AMPs were added to both vials A and B, along with the cross-linker (Di(ethylene glycol) diacrylate). The final component, methacrylic acid, was prepared in vial C by mixing the remainder of the extra added water/surfactant solution. All the vials were then shaken for 30 min to allow the solutions to mix thoroughly. Finally, vial C, followed by B and then A, were poured into a mold. Using an overhead mechanical stirrer, the solution was rapidly stirred for a controlled time period (20 s) in order to combine all the components before foaming and gelation occurred. The amounts of each component used are listed in Table 2. The porous hydrogels with Manuka honey (PH_MH) were prepared using the same method, with the Manuka honey added to the surfactant solution in vials A and B at two different concentrations (1 and 10% *w*/*w*).

### 4.3. Swelling Test

The swelling behaviour of the samples was investigated by completely immersing them in deionized water at room temperature. Next, the swollen hydrogels were removed and weighed at selected time intervals ranging from 1 to 30 min. Upon removal from the deionized water, the hydrogels were blotted to remove excess surface water before weighing. The swelling ratio (% swelling) was calculated based on the change in weight using the following equation:

(1) $$\%\;\mathrm{Swelling}=\frac{W_{f}-W_{i}}{W_{i}}\times 100\%$$

where *W_i_* and *W_f_* are initial weight and final weight at different times, respectively. The measurements were conducted three times for each sample and reported as the average % swelling percentage, with the standard deviation reported to indicate the level of uncertainty.

### 4.4. Surface Absorption

The surface absorption properties of all hydrogel samples were evaluated using a Dataphysics Model OCA20 (Filderstadt, Germany) contact angle apparatus. The surface absorption was found by plotting the drop volume (μL) vs. drop age (milliseconds). The OCA20 camera recorded at 36 frames per second, which allowed the drop volume to be accurately recorded during the absorption process, enabling the detailed, real-time surface absorption to be measured for each sample. The hydrogel samples were first cut into 10 mm sizes using a cork borer and placed on a glass sample holder. Then, a droplet of 10 µL of deionized water was deposited onto the surface of each hydrogel sample, and the software recorded the volume of liquid vs. drop age.

### 4.5. Morphological Observations

The morphology of the hydrogels was measured using scanning electron microscopy (SEM) (LEO Co., Cambridge, England, Model: 1455VP). The preparation of the samples for SEM were as follows: the hydrogel samples were cut into a diameter of 10 mm and placed on an aluminum stub. The hydrogels were then dehydrated in a desiccator in order to remove the moisture present in the hydrogel before coating it with gold. At this point, the hydrogels were ready for testing. The SEM images were used to measure the pore size, which was calculated using ImageJ software (version 2.3.0) and presented as average value.

The porosity of the hydrogel samples was assessed using SEM images and analyzed with the ImageJ software. The thresholding operation was utilized to distinguish between pores and solid material based on their pixel intensities. Pixels with intensities below a specified threshold value were identified as pores, while pixels with intensities above the threshold were categorized as solid materials. The porosity was calculated by dividing the area of the pores by the total area of the image, and the resulting value was multiplied by 100% to express it as a percentage. The porosity calculation formula can be expressed as:

(2) $$\%\;\mathrm{Porosity}=\frac{\mathrm{Area}\;\mathrm{of}\;\mathrm{pores}}{\mathrm{Total}\;\mathrm{area}}\times 100$$

### 4.6. Rheological Measurement

A rotational rheometer, ARES G2 (advanced rheometrics expansion system), TA Instrument, New Castle, DE, U.S.A was used to measure the viscoelastic behaviour of all the hydrogel samples. The samples were cut to a diameter of 25 mm using a cork borer and placed between two 25 mm serrated parallel plates with a 2–4 mm gap. The two test parameters consisted of a stain sweep from 0.01% to 150% at a constant frequency of 1 Hz and a frequency sweep from 0.1 to 25 Hz with 1% constant strain at 25 °C.

### 4.7. Cytotoxicity Test

The samples were cut into a cylinder shape with a diameter of 6 mm. Then, the samples were impregnated in 1 mL of serum-free Dulbecco’s Modified Eagle’s Medium (DMEM) Grand Island, NY, USA at room temperature for 24 h. At that time, the impregnated medium was sterilized using a syringe filter cap (0.2 µm). A suspension of normal human dermal fibroblast cells (NHDF cells) was placed in 96-well plates at a density of 1 × 10^4^ cells/well and incubated in DMEM containing 10% FBS, 1% penicillin, 1% streptomycin, and 0.1% amphotericin B at 37 °C in a CO_2_ incubator for 24 h. The medium was discarded and the NHDF cells were washed with PBS. Then, the cells were treated with the sterilized medium of the impregnated samples in each well. The untreated control was prepared using the NHDF cells with free-serum medium without the impregnation of samples. The cells were incubated at 37 °C in a CO_2_ incubator for 24 h and compared with the control group (untreated NHDF). After treatment, the cells were washed with PBS. Then, both groups were replaced with 200 μL of new free-serum medium. Then, 50 μL of XTT solution was added to each well and incubated for 4 h. The cell viability was determined by measuring the optical density (*OD*) at 490 nm using a microplate reader (EonTM, BioTek instrument, Winooski, VT, USA.) and equation 3. The *OD* values of each sample were calculated as the % viability and compared with 100% viability of the untreated cells:

(3) $$\mathrm{Cell}\;\mathrm{Viability}\;\left(\%\right)=\frac{OD_{S}}{OD_{C}}\times 100$$

where *OD_S_* is the absorbance of the samples and *OD_C_* is the control.

## Figures and Tables

**Table 1:** Average pore size and % porosity.

Samples	Average Pore Size (µm)	% Porosity
Porous hydrogel (PH)	108.5 ± 46.0	37.10 ± 34.5
Porous hydrogel with 1% Manuka Honey (PH_1% MH)	51.5 ± 24.2	21.73 ± 15.5
Porous hydrogel with 10% Manuka Honey (PH_10% MH)	50.5 ± 11.2	42.22 ± 11.1

**Table 2:** Chemical compositions of the hydrogel samples.

Sample	AMPs (g)	DI Water/F127 (g)	DI Water (g)	XL (g)	TEMED (g)	APS (g)	BA (g)	MAA (g)	MH (g)
Non-Porous hydrogel (NPH)	5.00	-	4.00	0.20	-	-	-	-	-
Porous hydrogel (PH)	5.00	4.00	-	0.20	0.65	0.65	0.50	0.25	-
Porous hydrogel with 1% Manuka honey (PH_1% MH)	5.00	3.90	-	0.20	0.65	0.65	0.50	0.25	0.1040
Porous hydrogel with 10% Manuka honey (PH_10% MH)	5.00	1.96	-	0.20	0.65	0.65	0.50	0.25	1.0400

## Data Availability

The raw/processed data required to reproduce these findings cannot be shared at this time as the data also forms part of an ongoing study.
